# Chronic spinal cord injury functionally repaired by direct implantation of encapsulated hair-follicle-associated pluripotent (HAP) stem cells in a mouse model: Potential for clinical regenerative medicine

**DOI:** 10.1371/journal.pone.0262755

**Published:** 2022-01-27

**Authors:** Koya Obara, Kyoumi Shirai, Yuko Hamada, Nobuko Arakawa, Michiko Yamane, Nanako Takaoka, Ryoichi Aki, Robert M. Hoffman, Yasuyuki Amoh

**Affiliations:** 1 Department of Dermatology, Kitasato University School of Medicine, Sagamihara, Kanagawa, Japan; 2 AntiCancer, Inc., San Diego, California, United States of America; 3 Department of Surgery, University of California San Diego, San Diego, California, United States of America; University of Minnesota Medical School, UNITED STATES

## Abstract

Chronic spinal cord injury (SCI) is a highly debilitating and recalcitrant disease with limited treatment options. Although various stem cell types have shown some clinical efficacy for injury repair they have not for SCI. Hair-follicle-associated pluripotent (HAP) stem cells have been shown to differentiate into neurons, Schwan cells, beating cardiomyocytes and many other type of cells, and have effectively regenerated acute spinal cord injury in mouse models. In the present report, HAP stem cells from C57BL/6J mice, encapsulated in polyvinylidene fluoride membranes (PFM), were implanted into the severed thoracic spinal cord of C57BL/6J or athymic nude mice in the early chronic phase. After implantation, HAP stem cells differentiated to neurons, astrocytes and oligodendrocytes in the regenerated thoracic spinal cord of C57BL/6J and nude mice. Quantitative motor function analysis, with the Basso Mouse Scale for Locomotion (BMS) score, demonstrated a significant functional improvement in the HAP-stem-cell-implanted mice, compared to non-implanted mice. HAP stem cells have critical advantages over other stem cells: they do not develop teratomas; do not loose differentiation ability when cryopreserved and thus are bankable; are autologous, readily obtained from anyone; and do not require genetic manipulation. HAP stem cells therefore have greater clinical potential for SCI repair than induced pluripotent stem cells (iPSCs), neuronal stem cells (NSCs)/neural progenitor cells (NPCs) or embryonic stem cells (ESCs). The present report demonstrates future clinical potential of HAP-stem-cell repair of chronic spinal cord injury, currently a recalcitrant disease.

## Introduction

Spinal cord injury (SCI) is a severely debilitating and recalcitrant condition leading to neurological dysfunction, loss of independence, respiratory failure, psychological morbidities, and an increased mortality rate. There are approximately 500,000 new case of SCI each year world-wide [1, 2]. There is no effective treatment option to repair the injured spinal cord and restore lost function, including walking ability [2]. A variety of stem cells have been used to attempt to regenerate SCI, with only limited success [3, 4]. Previously, we discovered nestin-expressing stem cells in the bulge area of the hair follicle [5, 6]. We termed these cells hair-follicle-associated pluripotent (HAP) stem cells. HAP stem cells from both mouse and human have multilineage differentiation capacity that could produce neurons, glia, smooth muscle cells, melanocytes, keratinocytes, cardiac muscle cells and dopaminergic neurons [7–11].

HAP stem cells from mice were previously used to repair the severed sciatic nerve in mouse models. HAP stem cells implanted into the gap region of a severed sciatic nerve in mice enhanced regeneration and the restoration of nerve function and walking ability. The implanted HAP stem cells transdifferentiated largely into Schwann cells [12]. Human HAP stem cells also were previously implanted in the severed sciatic nerve of mice and differentiated into glial fibrillary-acidic-protein (GFAP)-positive Schwann cells and enhanced the recovery of pre-existing axons, resulting in nerve generation and functional recovery [13]. HAP stem cells from mice have been also able to repair the severed spinal cord in mouse models. In subsequent previous experiments, the thoracic region of spinal cord of C57BL/6 immunocompetent mice was severed and implanted HAP stem cells effected repair of the severed site and restored walking function. HAP stem cells implanted in the severed spinal cord formed Schwann cells which facilitated repair of the severed spinal cord. The spinal cord rejoined by HAP stem cells regained comprehensive hind-limb locomotor performance [14, 15]. Recently we have shown that HAP stem cells encapsulated in polyvinylidene fluoride membranes (PFM) efficiently effected regeneration of the severed sciatic nerve and spinal cord in the acute phase in the C57BL/6J mouse model [16, 17].

In the present study, we demonstrate that mouse HAP stem cells, encapsulated in PFM, effected structural and functional regeneration of SCI in the early chronic phase when implanted in the injured spinal cord in mouse models. The potential clinical advantages of HAP stem cells to regenerate chronic SCI are discussed.

## Materials and methods

### Mice

Transgenic C57BL/6J-EGFPmice (GFP mice) were obtained from the Research Institute for Microbial Diseases (Osaka University, Osaka, Japan) [18]. C57BL/6J mice and BALB/cAJcl-nu/nu mice (nude mice) were obtained from CLEA Japan (Tokyo, Japan). All procedures involving animals complied with the guidelines of the US National Institutes of Health and were approved by the Animal Experimentation and Ethics Committees of the Kitasato University School of Medicine. All efforts were made to minimize animal suffering and reduce the number of animals used. The method of euthanasia at the end of the experiment was cervical dislocation.

### Isolation, culture and encapsulation of HAP stem cells

**Fig 1:**
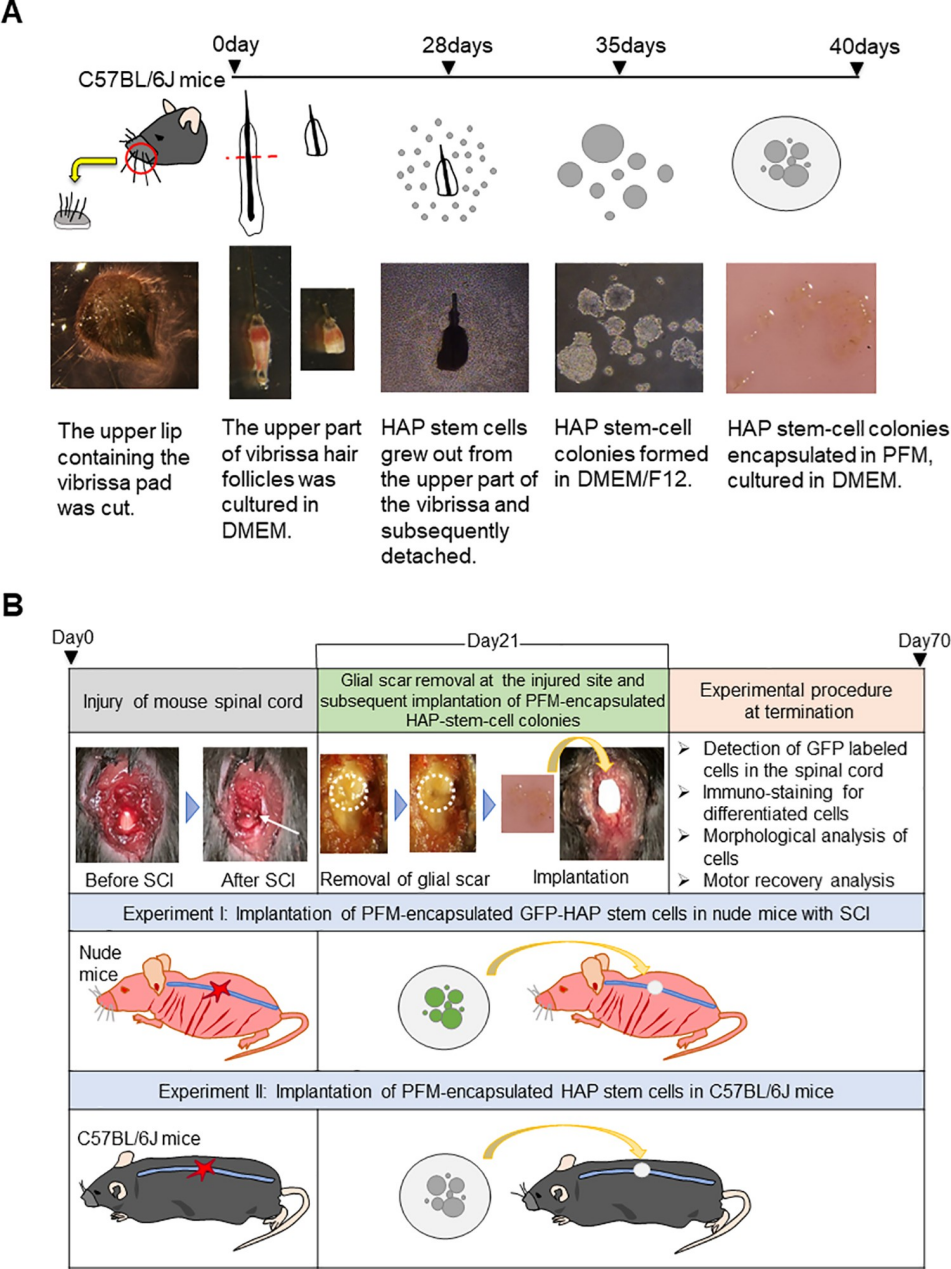
Experimental flow diagram. (A) Procedure for isolation, culture and encapsulation of HAP stem cells. (B) Procedure for implantation of PFM-encapsulated HAP stem cells. Experiment I: GFP-expressing PFM-encapsulated HAP stem cells from C57BL/6J GFP mice were implanted in the severed thoracic spinal cord of nude mice with SCI three week after injury. Experiment II: PFM-encapsulated HAP stem cells of C57BL/6J mice without GFP expression were implanted in C57BL/6J mice with SCI.

Vibrissa hair follicles were resected from green-fluorescent-protein (GFP) expressing transgenic or non-GFP C57BL/6J mice as described previously [7] (Fig 1A). To obtain the vibrissa follicles from mice, the animals were anesthetized with a combination anesthetic of 0.75 mg/kg medetomidine, 4.0 mg/kg midazolam and 5.0 mg/kg butorphanol [19]. The upper lip, containing the vibrissa pad, was cut and its inner surface exposed. Intact vibrissa follicles were dissected under a binocular microscope. The vibrissae from the pad were plucked by pulling them gently with a fine forceps. The upper part of the vibrissa follicles was separated as described previously [9]. The upper parts of the isolated vibrissa hair follicles were cultured in DMEM (Sigma Aldrich, St. Louis, MO, USA), containing 10% fetal bovine serum (FBS), 50 μg/ml gentamycin (GIBCO, Grand Island, NY, USA), 2 mM L-glutamine (GIBCO) and 10 mM HEPES (MP Biomedicals, Solon, OH, USA) for four weeks. The growing GFP or non-GFP HAP stem cells were detached and transferred to non-adhesive cell-culture dishes with DMEM/F12 (GIBCO), containing 2% B-27 (GIBCO) and 5 ng/ml basic fibroblast growth factor (bFGF) (Millipore, Temecula, CA, USA). After one week culture, the HAP stem cells formed colonies [7, 8]. HAP stem cell colonies were subsequently transferred and cultured on sterilized PFM (Millipore, Darmstadt, Germany) in DMEM for 5 days (Fig 1A). There were 1.3 x 10^5^ cells on the PFM for implantation. To confirm their differentiation with immunostaining, GFP HAP stem cell colonies were cultured in Lab-Tek chamber slides (Nunc, Rochester, NY, USA) with DMEM for two weeks.

### Spinal cord injury (SCI) mouse model

Thoracic SCI was performed as described previously [17]: the thoracic spinal cord of nude or C57BL/6J mice was severed under anesthesia. The dorsal side of the thoracic vertebra of the mice was exposed, and a longitudinal incision of approximately 1.0 cm was made to the median. The tenth thoracic vertebral arch was resected with scissors, and the dural membrane of the thoracic spinal cord was exposed. To effect SCI, the thoracic spinal cord was pressed with an 18G needle with a weight of 20 g for 20 min. The paravertebral muscles, superficial fascia and skin were then sutured, respectively. The incision was closed with 6–0 nylon sutures (SIGMA REX, Tokyo, Japan). All injured mice received manual bladder evacuation once every two days until recovery of function.

### Implantation of encapsulated HAP stem cells in the early chronic phase of SCI

At 21 days post-SCI, the severed thoracic spinal cord in the nude or C57BL/6J mice was exposed under anesthesia and the glial scar in the injured spinal cord was removed with a 26G needle. HAP stem cell colonies, encapsulated on PFM, were cut into discs of 3 mm diameter and implanted into the thoracic spinal cord where the glial scar was removed (Fig 1B). The glial scar in the control mice, without implantation, was also removed. The surgical skin wound was closed as described above. Mice that died during the course of the experiment were excluded from the evaluation.

### Histological analysis

Immunostaining was performed as described previously [17]: HAP stem cell colonies were cultured on Lab-Tek chamber slides, were incubated with the following antibodies: anti-βIII tubulin mouse monoclonal antibody (1:500, Tuj1clone; Covance, CA, USA); or anti-glial fibrillary acidic protein (GFAP) mouse monoclonal antibody (1:200; LAB VISION, CA, USA). The HAP stem cells were then incubated with goat anti-mouse IgG conjugated with Alexa Flour 568^®^ (1:400, Molecular Probes, OR, USA) and 4’,6-diamino-2-phenylindole, dihydrochloride (DAPI) (Molecular Probes). At 70 days post-SCI and 49 days post-HAP-stem-cell implantation, the thoracic spinal cord was directly observed by fluorescence microscopy. The nerve samples were then excised and frozen blocks and frozen sections were made. Frozen-sections were incubated with anti-βIII tubulin, anti-GFAP, anti-myelin basic protein (MBP) rabbit monoclonal antibody (1:200, Chemicon, Temecula, CA, USA) and anti-Iba-1 mouse monoclonal antibody (1:500, FUJIFILM Wako, Tokyo, Japan) then were incubated with goat anti-mouse IgG conjugated with Alexa Flour 568^®^ and DAPI. The excised nerve samples were also formalin-fixed and paraffin-embedded-blocks (FFPB) were made. The FFPB sections on slides were stained with hematoxylin and eosin (H&E), or immunostained. For immunostaining, FFPB sections on slides were incubated with anti-βIII tubulin and anti-GFAP and then were treated with Dako ChemMate Envision kit/HRP (Dako Japan, Tokyo, Japan). The sections were developed with 3,3’-diaminobenzidine tetrachloride (DAB) (Dako) and then incubated with Mayer’s hematoxylin solution. Immunostaining for βIII tubulin, GFAP, MBP and Iba-1 was then performed, in the control and repaired spinal cord, in sagittal sections and observed at 100× magnification. The βIII tubulin-, GFAP-, MBP- and Iba-1-positive areas were quantified in the area at the lesion epicenter, and images were captured at 100× magnification, from just below the dural membrane. One section per mouse was used for each analysis. Quantitative analyses were performed using ImageJ software (version 1.52; National Institutes of Health, USA) as previously described [20–22]. The threshold values were maintained at a constant level for all analyses. The diameter of the control and HAP-stem-cell repaired spinal cord was quantified using sagittal sections from the lesion epicenter and 0.5, 1.0 mm rostral and 0.5, 1.0 mm caudal to the epicenter with images, captured at 40x magnification.

### Motor function analysis

Motor function analysis was performed as described previously [17]: The mice were randomly assigned to the implanted group and the non-implanted group. Control and HAP-stem cell-treated mice were observed for motor function in a transparent case (30 × 30 cm), weekly up to 70 days post SCI, using the Basso Mouse Scale for Locomotion (BMS) score [23].

### Statistical analysis

All experimental data are expressed as the mean ± SEM. An unpaired the Student’s *t*-test was used to evaluate the differences between groups with histological analysis regarding sectional spinal area with HE staining and βIII tubulin-, GFAP-, MBP-, Iba-1-positive area. Two-way ANOVA followed by the Bonferroni post hoc test was used to examine the differences between groups in assessments of the BMS analysis. A probability value of *P ≤* 0.05 is considered significant.

## Results

### Differentiation of HAP stem cell colonies into neurons and glial cells on polyvinylidene fluoride membranes (PFM) in culture

**Fig 2:**
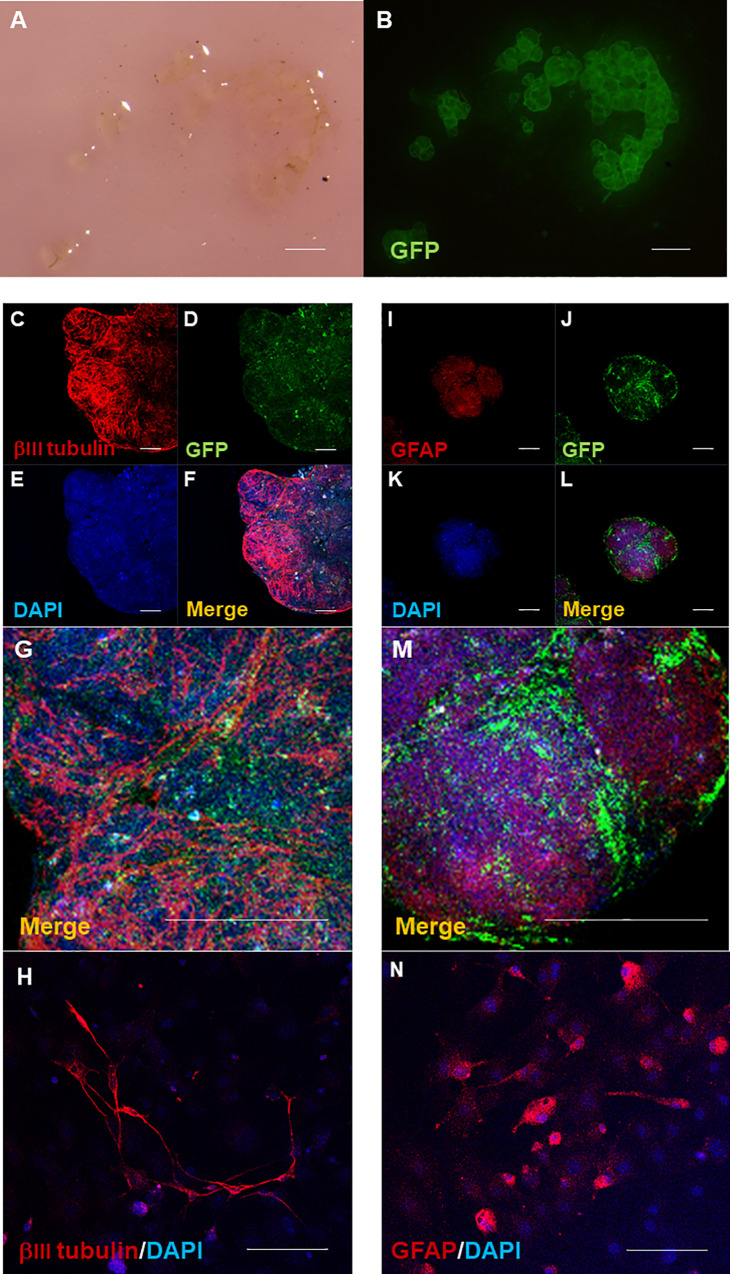
Differentiation of PFM-encapsulated HAP-stem-cell colonies. Stereomicroscopy shows HAP stem cell colonies encapsulated and aggregated at the center of the PFM, expressing GFP (A, B). Bar = 500 μm. Immunofluorescence staining shows that the PFM-encapsulated HAP stem cells differentiated into neurons (C-H) and glial cells. (I-N) In the encapsulated HAP stem cell colonies, neurons have a fibrous shape (H) and glia cells have a round to oval cytoplasm with several protrusions around it (N). Red = βIII tubulin or GFAP; Blue = DAPI; Green = GFP. Bar = 100 μm.

Stereomicroscopy shows HAP stem cell colonies became encapsulated and aggregated on the center of the PFM (Fig 2A). Fluorescence microscopy shows GFP-expressing PFM-encapsulated HAP stem cells growing as colonies in culture (Fig 2B). Immunofluorescence staining shows that the PFM-encapsulated HAP stem cells differentiated into neurons (Fig 2C–2H) and glial cells (Fig 2I–2N) at five and 14 days of culture, respectively. Both neurons and glial cells proliferated uniformly throughout the aggregated HAP stem cell-colonies. In the PFM-encapsulated HAP stem cell colonies, neurons had a fibrous shape (Fig 2H) and glia cells had a round to oval cytoplasm with several protrusions around them (Fig 2N).

### PFM-encapsulated HAP stem cells differentiate into neurons, astrocytes and oligodendrocytes in the severed spinal cord after implantation at the early chronic phase

**Fig 3:**
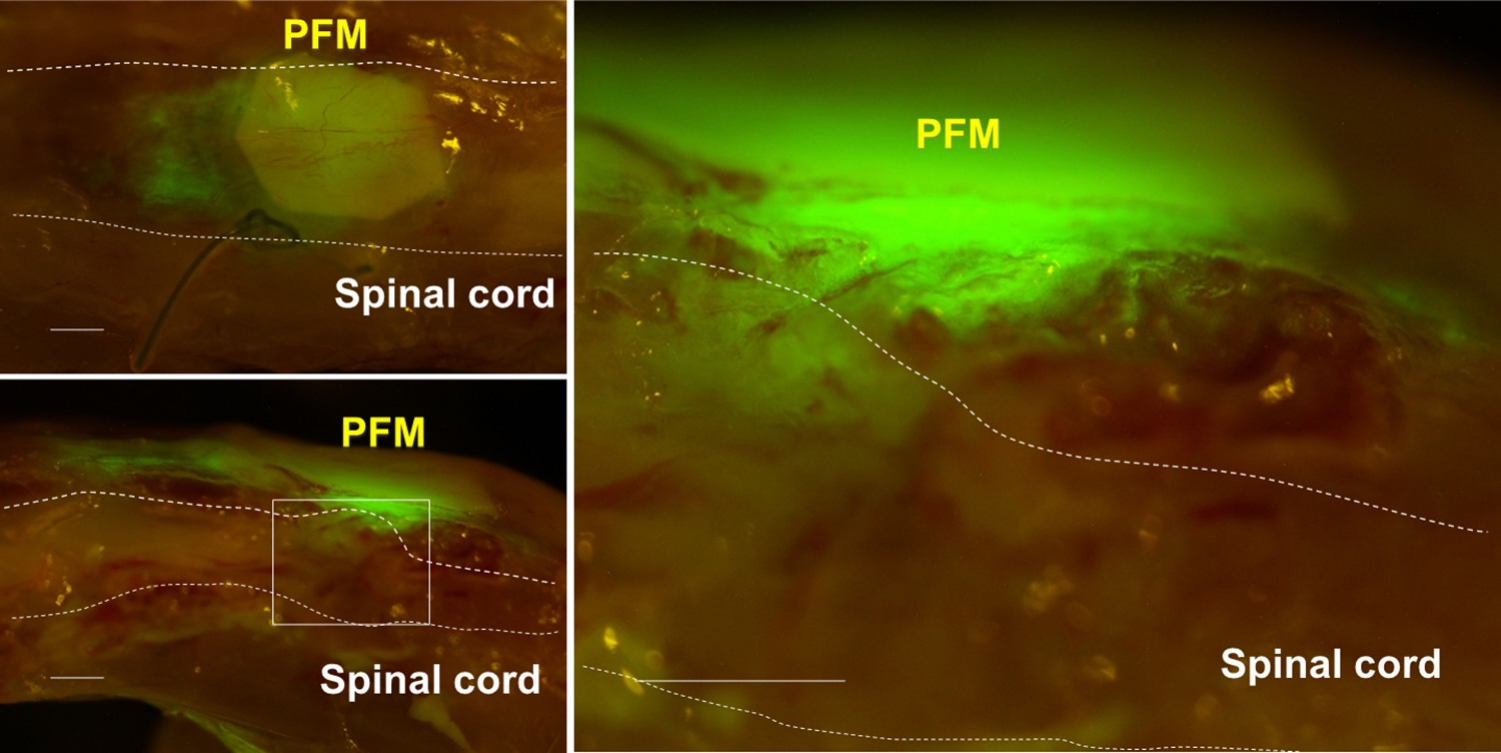
Expansion of implanted HAP stem cells in the severed spinal cord. GFP-expressing HAP stem cells migrated out from the PFM and joined the severed thoracic spinal cord. Left panel = low-magnification of coronal section (upper) and sagittal section (lower). Right panel = high-magnification of white boxed area. Bar = 500 μm.

At 70 days post SCI and 49 days post-HAP-stem-cell implantation, the repaired thoracic spinal cord in nude mice was directly observed by fluorescence microscopy which showed that GFP-expressing HAP stem cells migrated out from the PFM and joined the severed thoracic spinal cord (Fig 3).

**Fig 4:**
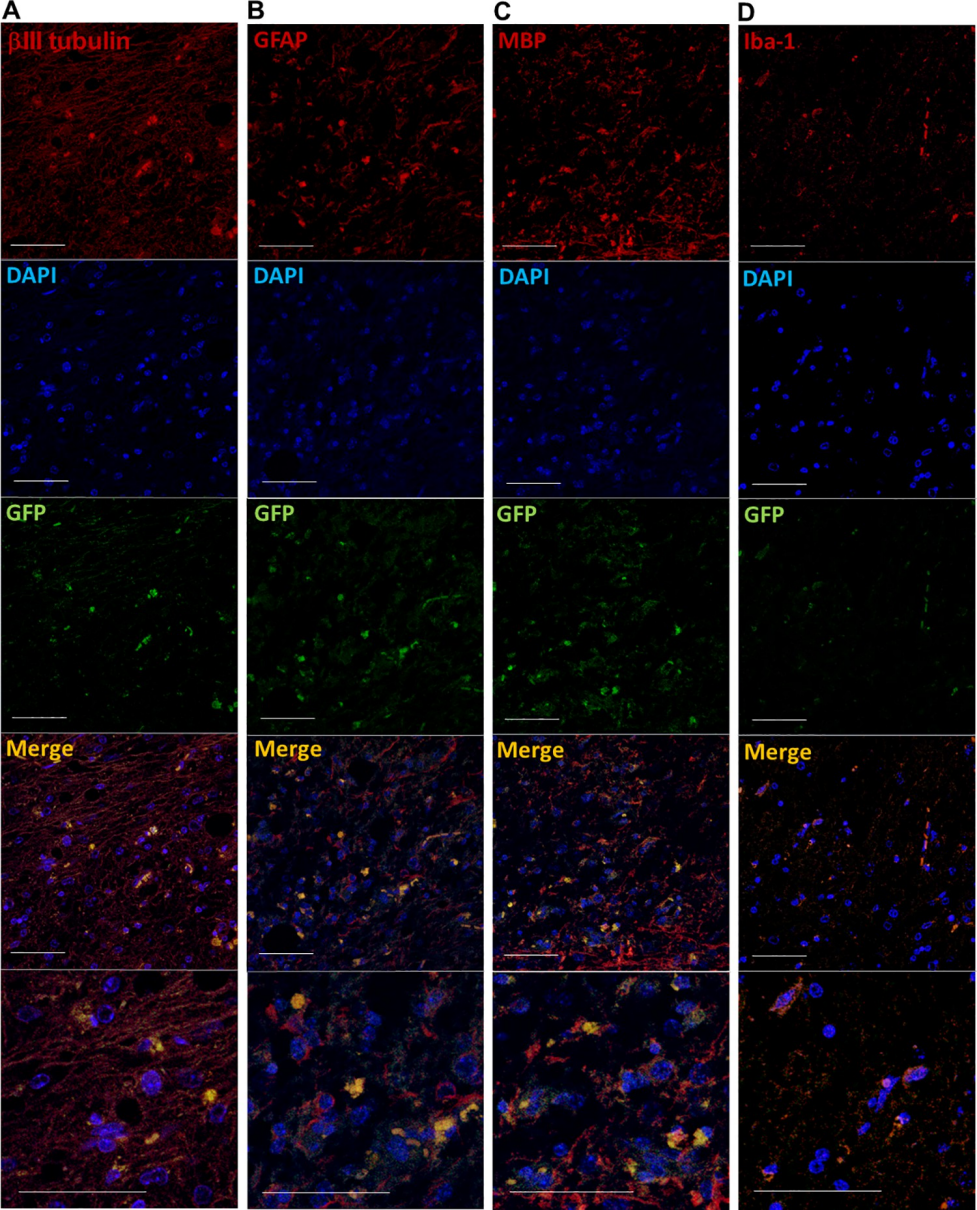
Immunofluorescence staining of the severed spinal cord implanted with HAP stem cells. Immunofluorescence staining shows that in the joined area of the previously severed spinal cord, the implanted HAP stem cells differentiated to neurons (A), astrocytes (B), oligodendrocytes (C). Microglia were GFP negative in the joined area of severed spinal cord (D). Red = βIII tubulin (A), GFAP (B), MBP (C) or Iba-1 (D); Green = GFP (A-D); Blue = DAPI (A-D); Merged (A-D). Bar = 50 μm. All images show sagittal sections of the spinal cord.

Nude mice were used in this experiment in order to readily visualize the GFP-expressing HAP stem cells in the regenerated spinal cord. Immunofluorescence staining shows that the implanted HAP stem cells differentiated into neurons, astrocytes and oligodendrocytes (Fig 4A–4C), which effected joining of the severed spinal cord. Microglia infiltrated slightly in the joined area of severed spinal cord and were negative for GFP indicating they were not of HAP stem-cell origin (Fig 4D).

**Fig 5:**
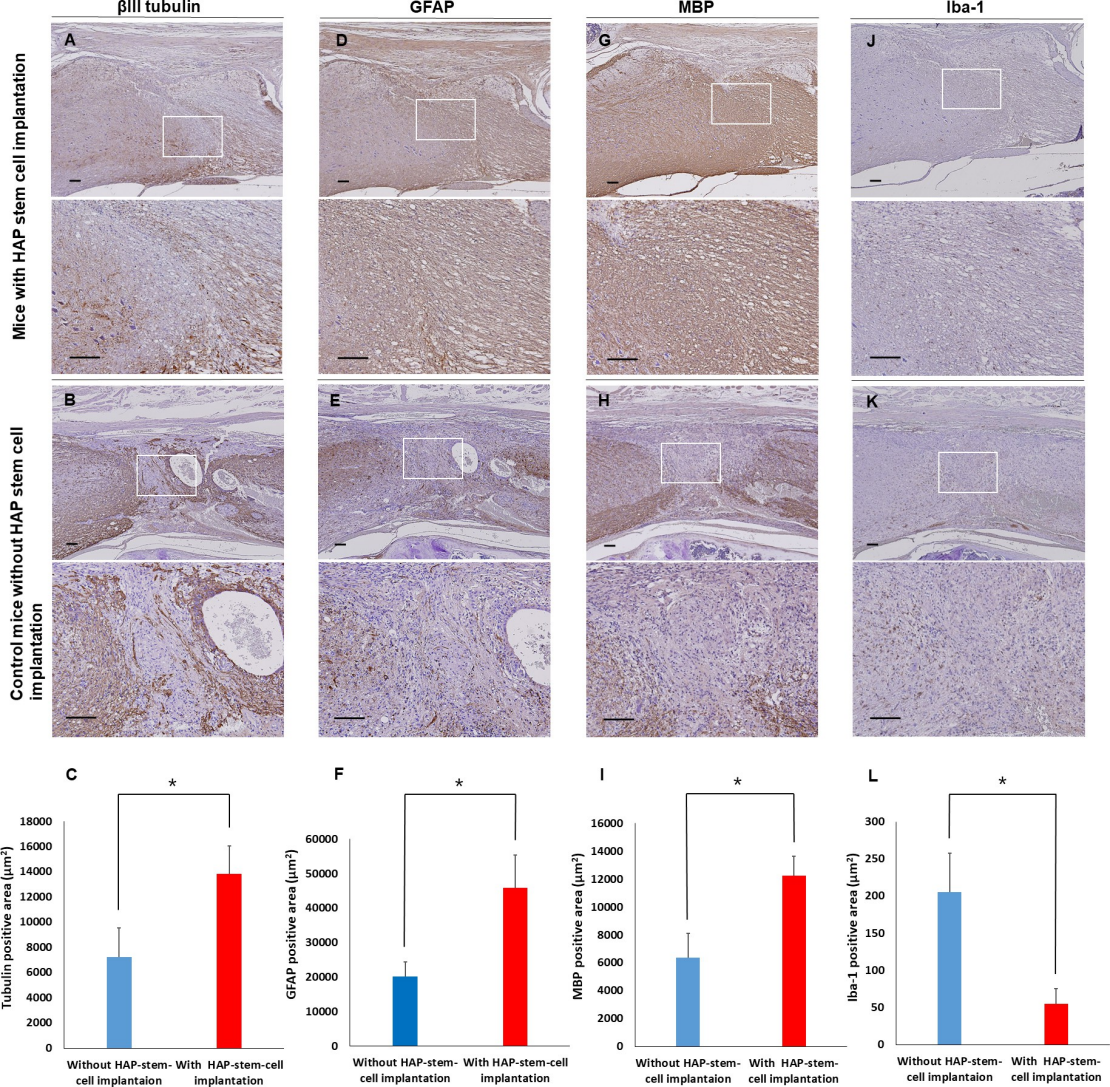
Immunostaining of the severed spinal cord implanted with HAP stem cells. (A, B) βIII tubulin expression in the repaired spinal cord after HAP-stem-cell implantation and without implantation. (C) The βIII tubulin-positive area in sagittal sections in mice with implanted HAP stem cells and control mice (mice with HAP-stem-cell implantation: n = 5; control mice without implantation: n = 5). *P < 0.05. (D, E) GFAP expression in the repaired spinal cord after HAP stem cell implantation and without implantation. (F) The GFAP-positive area in sagittal sections in the mice with implanted HAP stem cells and control mice (mice with HAP-stem-cell implantation: n = 5; control mice without implantation: n = 5). *P < 0.05. (G, H) MBP expression in the repaired spinal cord after HAP-stem-cell implantation and in the un-repaired spinal cord without implantation. (I) The MBP-positive area in sagittal sections in the mice with implanted HAP stem cells and control mice with an un-repaired spinal cord (mice with HAP-stem-cell implantation: n = 5; control mice without implantation: n = 5). *P < 0.05. (J, K) Iba-1 expression in the repaired spinal cord after HAP-stem-cell implantation and in the un-repaired spinal cord without implantation. (F) The Iba-1-positive area in sagittal sections in the mice with implanted HAP stem cells and control mice without HAP-stem-cell implantation (mice with HAP-stem-cell implantation: n = 5; control mice without implantation: n = 5). *P < 0.05. Upper panels = low-magnification. Bar = 100 μm. Lower panels = high-magnification of white boxed area. Bar = 100 μm. All images show sagittal sections with the spinal cord.

Immunostaining showed that HAP stem cells implanted in the severed part of the spinal cord of C57BL/6J mice also differentiated to neurons, astrocytes and oligodendrocytes (Fig 5A, 5D and 5G). In the un-implanted control mice, neurons, astrocytes and oligodendrocytes did not increase in the severed part of the spinal cord (Fig 5B, 5E and 5H). There were less microglia in the severed spinal cord implanted with HAP stem cells than in the un-implanted control mice (Fig 5J and 5K). A greater number of βIII tubulin-positive neurons, GFAP-positive astrocytes and MBP-positive oligodendrocytes were present in the spinal cord of mice implanted with HAP stem cells than in spinal cord of the un-implanted control mice (*P* < 0.05) (Fig 5C, 5F and 5I). There were significantly less Iba-1-positive microglia in the spinal cord implanted with HAP stem cells than in the spinal cord of un-implanted control mice (*P* < 0.05) (Fig 5L).

### PFM-encapsulated HAP stem cells suppress scar formation and atrophy in the severed spinal cord after implantation during the early chronic phase

**Fig 6:**
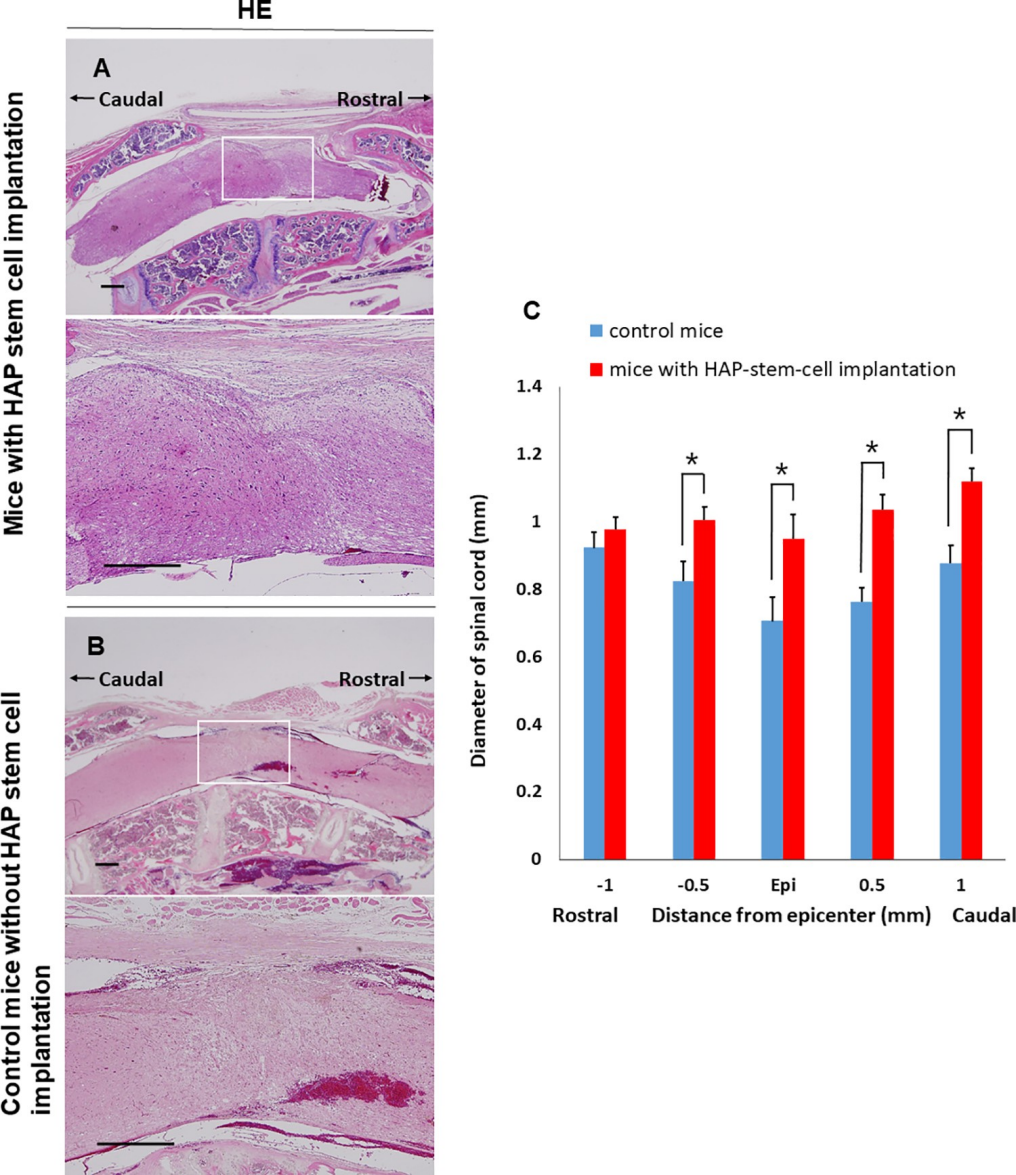
H&E staining of the severed spinal cord implanted with HAP stem cells. (A) HAP stem-cell-implantation rejoined the severed spinal cord of mice. (B) Control mice without implantation showed granulation tissue in the severed spinal cord. (C) Diameter of the severed spinal cord after HAP-stem-cell implantation (mice with HAP-stem-cell implantation: n = 5; control mice without implantation: n = 5). *P < 0.05. Upper panels = low-magnification. Bar = 500 μm. Lower panels = high-magnification of white boxed area. Bar = 500 μm. All images show sagittal sections with the spinal cord.

H&E staining showed that many spindle-like cells grew in the severed part of the thoracic spinal cord in C57BL/6J mice implanted with HAP stem cells (Fig 6A). No tumors were found in the spinal cord after implantation with HAP stem cells. H&E staining of the mice without HAP-stem-cell implantation showed granulation tissue in the severed part of the thoracic spinal cord (Fig 6B). The diameter of spinal cord was significantly larger in the C57BL/6J mice with implanted HAP stem cells compared to the untreated-control mice (spinal cord with implanted HAP stem cells vs. control: *P* < 0.05 at 0.5 mm rostral to epicenter; *P* < 0.05 at epicenter; *P* < 0.05 at 0.5 mm caudal to epicenter; *P* < 0.05 at 1.0 mm caudal to epicenter) (Fig 6C).

### Mice with HAP-stem-cell repaired spinal cord injury at the early chronic phase regain motor function

**Fig 7:**
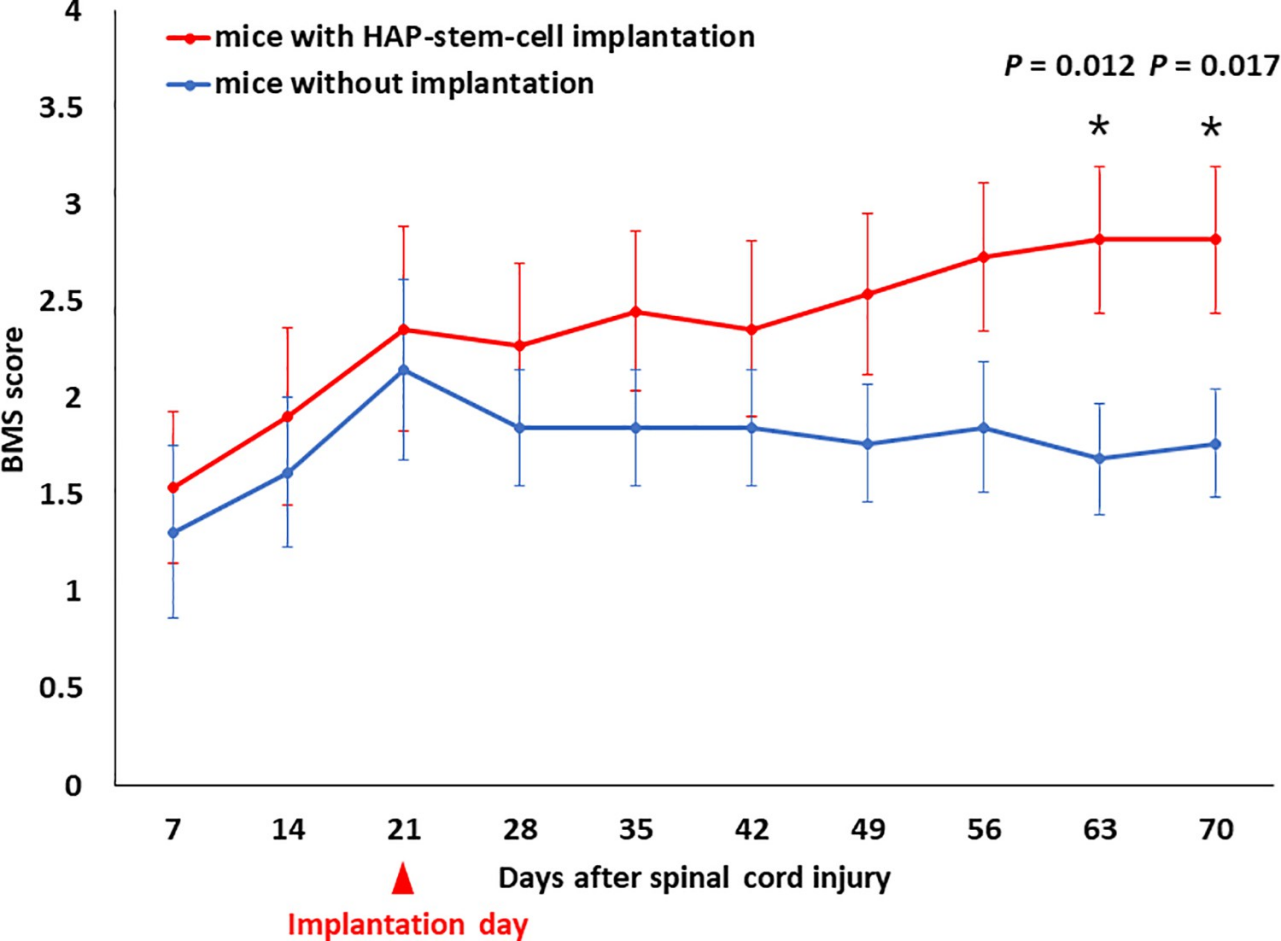
Motor function analysis of HAP-stem-cell implanted mice. Basso Mouse Scale for Locomotion (BMS) score for each group over the 70 days post-SCI. Although there was no significant difference in the BMS score among mice implanted with HAP stem cells and mice without HAP-stem-cell implantation until day 56, mice implanted with HAP stem cells exhibited significantly better functional recovery than mice without HAP-stem-cell implantation on day 63 and thereafter. (mice with HAP-stem-cell implantation: n = 11; control mice without implantation: n = 13). *P < 0.05.

Motor function was assessed weekly with BMS scoring up to 70 days post SCI and 49 days post HAP-stem-cell implantation in C57BL/6J mice. The motor function of mice with implanted HAP stem cells had a higher BMS score significantly than the un-implanted control mice at day 63 and 70 (day 63: *P* = 0.012; day 70: *P* = 0.017) (Fig 7). There was a slight but not significant difference in BMS score before and at the time point of implantation between the two groups.

## Discussion

Chronic spinal cord injury (SCI) is a recalcitrant disease that greatly reduces the quality of life. Stem cells of various types, including neuronal stem cells (NSCs)/ neural progenitor cells (NPCs) have been used for repair of SCI in animal models and the clinic with limited success [24]. For example, Rosenzweig et al. grafted human spinal cord NPCs into rhesus monkeys after SCI, where they expressed neuronal and glial markers [25]. Curtis et al. have tested NSC (NSI-566) in SCI patients in a Phase I and found this treatment is safe [26, 27]. The NPCs were not autologous and required immunosuppression for transplantation. For example, NPCs derived from induced pluripotent stem cells (iPSC-NPCs) were implanted in SCI minipigs where they differentiated into neurons and glial cells, but engraftment of the allogeneic iPSC-NPCs required immunosuppression [28]. The problem with iPSCs is that can form teratomas [29–31]. HAP stem cells used in the present study are readily accessible from everyone, and can be used autologously without immune-suppression, do not form tumors, and can be cryopreserved without loss of pluripotency, allowing individualized banking [7, 32, 33].

The present study suggests HAP stem cells may have greater potential than other stem cells for spinal cord regeneration in the chronic phase after injury. We have already demonstrated the efficacy of HAP stem cells implantation for SCI in the acute phase [17] and the present study shows that HAP stem cells are effective for early chronic-phase SCI. The present study shows that HAP stem cells implanted in the severed spinal cord of nude mice proliferate and differentiate into neurons, astrocytes and oligodendrocytes. Autologous implantation of HAP stem cells into the severed spinal cord of C57BL/6J mice resulted the significant proliferation of neurons, astrocytes and oligodendrocytes, and suppression of spinal cord atrophy. HAP stem cells, without neural induction, survived and differentiated into three neural lineage cells in the severed spinal cord, including neurons, astrocytes and oligodendrocytes. All et al. reported the effectiveness of hiPSC-derived oligodendrocyte progenitors (OPs) transplantation for SCI in rats [34]. However, these cells predominantly differentiated into oligodendrocytes, and less so into neurons. Graft-derived neurons are expected to play a significant role in improving functional recovery after transplantation. Kawabata et al. reported the benefits of transplanting human-iPSC derived oligodendrocyte precursor cell- enriched NSCs/NPCs in a SCI model. The difficulty in using these cells is that they required neural induction [35].

We further demonstrated that microglia are reduced in the severed spinal cord implanted with HAP stem cells. Microglia which are the innate immune cells in the central nervous systems, are of vital importance for the repair of SCI [36]. Infiltrative microglia found during inflammation are classified into M1 type, which acts on functionally contradictory injuries (inflammatory), and M2 type, which acts on protective (anti-inflammatory) [37]. During development of SCI, M2 polarized microglia were able to remove apoptotic cells and inappropriate neural connections, whereas M1 polarized microglia in the injured spinal cord and generated various pro‐inflammatory cytokines, such as TNF-α, IL‐1β and IL‐6, which aggravate secondary injury after SCI [38, 39]. In the present study, decreasing microglia in the spinal cord may have been resulted with HAP stem cell implantation promoting improvement of the microenvironment and regeneration of the severed spinal cord.

The timing of stem cell transplantation is important for successful functional recovery of the damaged spinal cord. In the chronic stage after SCI, glial scars form at the injured site and inhibit the regeneration of neuronal axons. Okano et al. reported that the optimal timing of transplantation of iPSC is 1–2 weeks after SCI [40]. Most studies targeting chronically injured spinal cord have reported no significant recovery of function. Kumamaru et al. indicated that failure to regenerate chronic SCI is not due to the lack of therapeutic activities of engrafted NSCs/NPCs but the refractory state of the chronically injured spinal cord [4]. Nisimura et al. showed that to achieve functional recovery by NSCs/NPCs transplantation for chronic SCI, modification of the microenvironment of the injured spinal cord, focusing on glial scar formation and the inflammatory phenotype, should be considered [41]. However, Okano et al. suggested that rehabilitative treatment represents a therapeutic option for locomotor recovery after NS/PC transplantation in chronic SCI [42]. In contrast, HAP stem cells alone improved the walking function of mice with early chronic SCI as shown in the present study.

In conclusion, the results of the present study demonstrated that HAP stem cells are capable of surviving and differentiating when implanted in the injured microenvironment at the early chronic phase. HAP stem cells proliferated rostrally from the implantation site in the spinal cord which is important for recovery. The present study demonstrated HAP stem cells are also capable of enhancing locomotor recovery in the early chronic phase of spinal cord injury. We previously showed that encapsulated cultured HAP stem cells in PFM membranes effected acute SCI regeneration and that empty PFM membranes were not effective [17]. Therefore, encapsulated HAP stem cells in PFM membranes used in the present study have important potential to be an effective future clinical strategy for regeneration of chronic spinal cord injury.

## Supporting information

S1 Fig Original immunofluorescence images. The separate channels for each image are shown without alterations of contrast or brightness and no cropping (Scale bar = 50μm).
(PDF)

S1 Table Raw data used for graphs and calculations. (XLSX)
