# Supplementary figures and images for: Chronic spinal cord injury functionally repaired by direct implantation of encapsulated hair-follicle-associated pluripotent (HAP) stem cells in a mouse model: Potential for clinical regenerative medicine
Source: PLoS One. 2022 Jan 27;17(1):e0262755. doi: 10.1371/journal.pone.0262755 (PMC8794105; doi:10.1371/journal.pone.0262755)

Figure4A

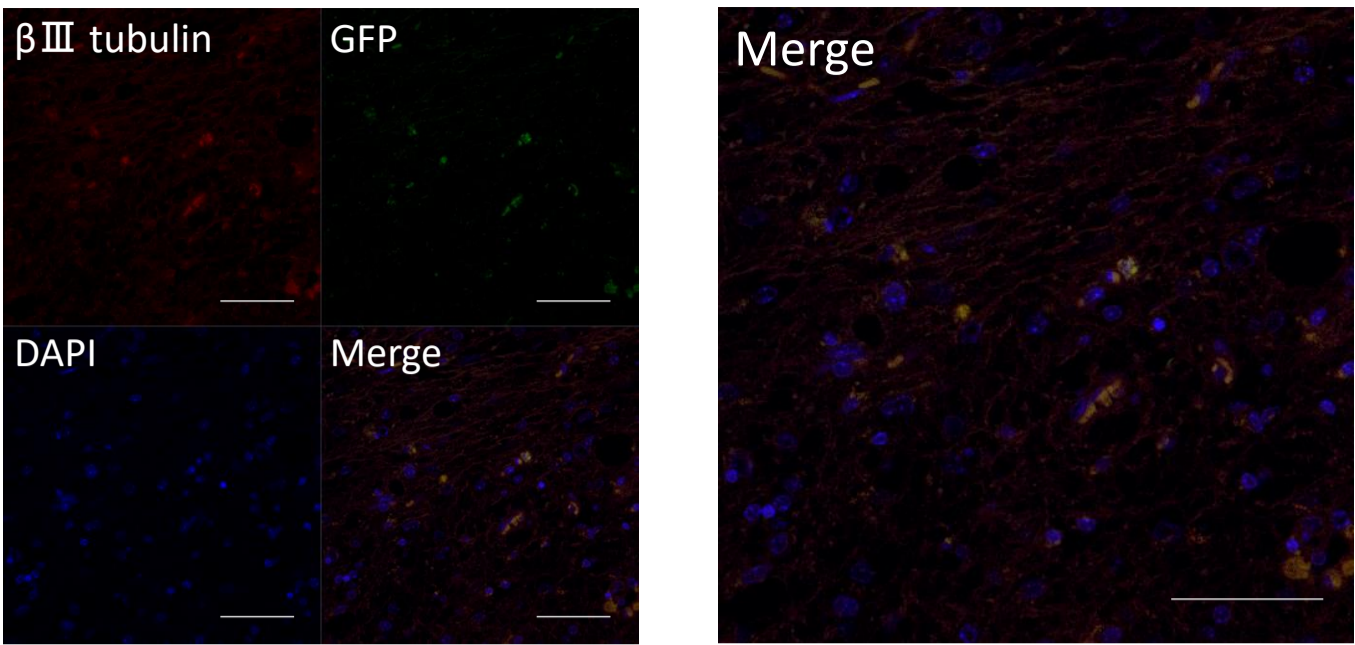

Figure4B

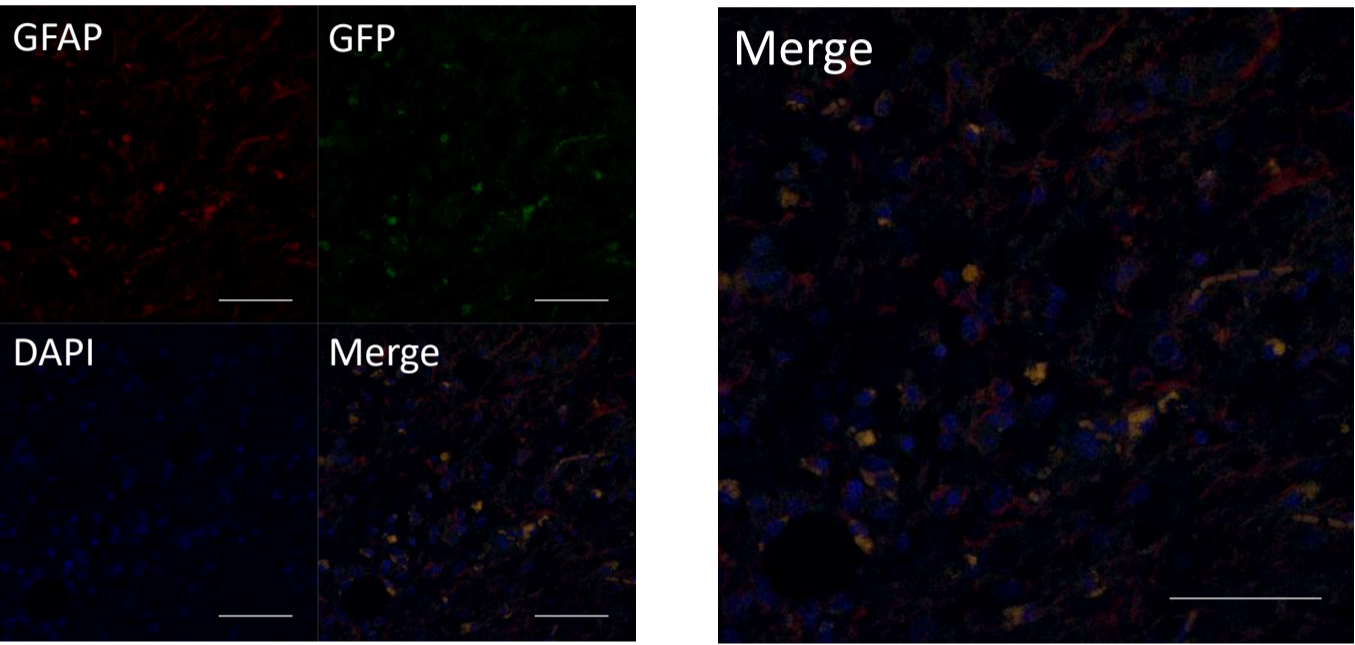

Figure4C

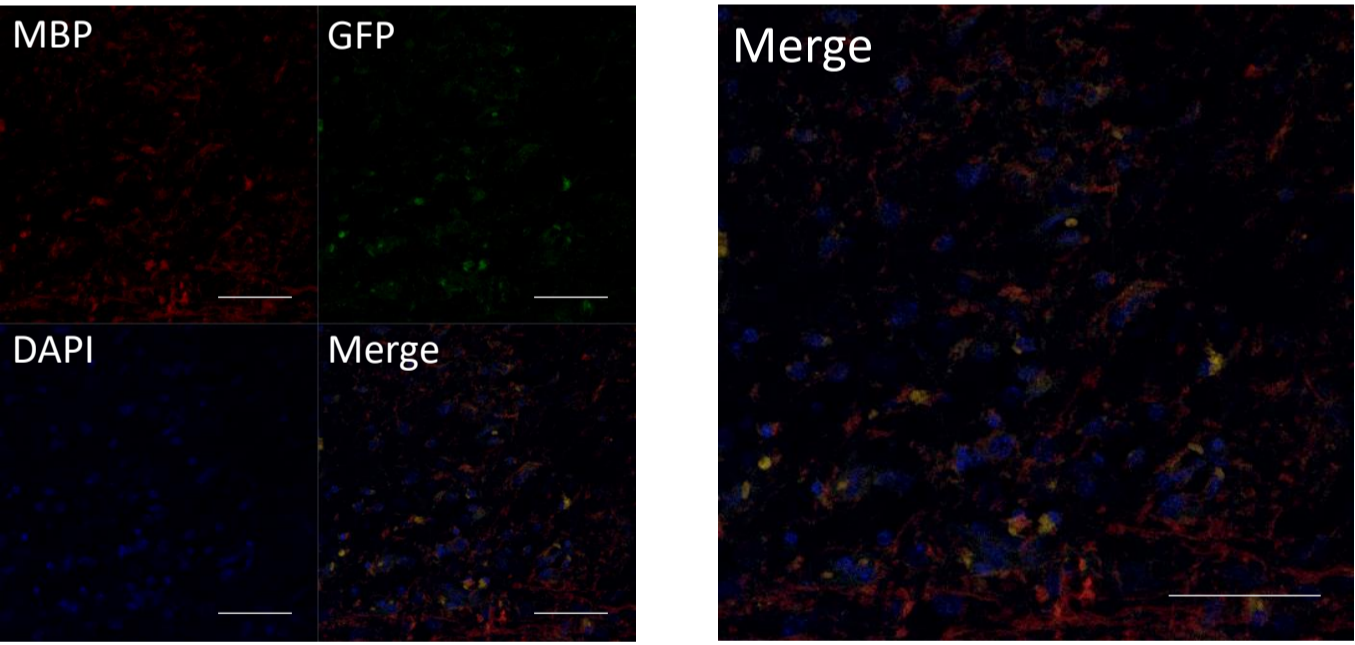

Figure4D

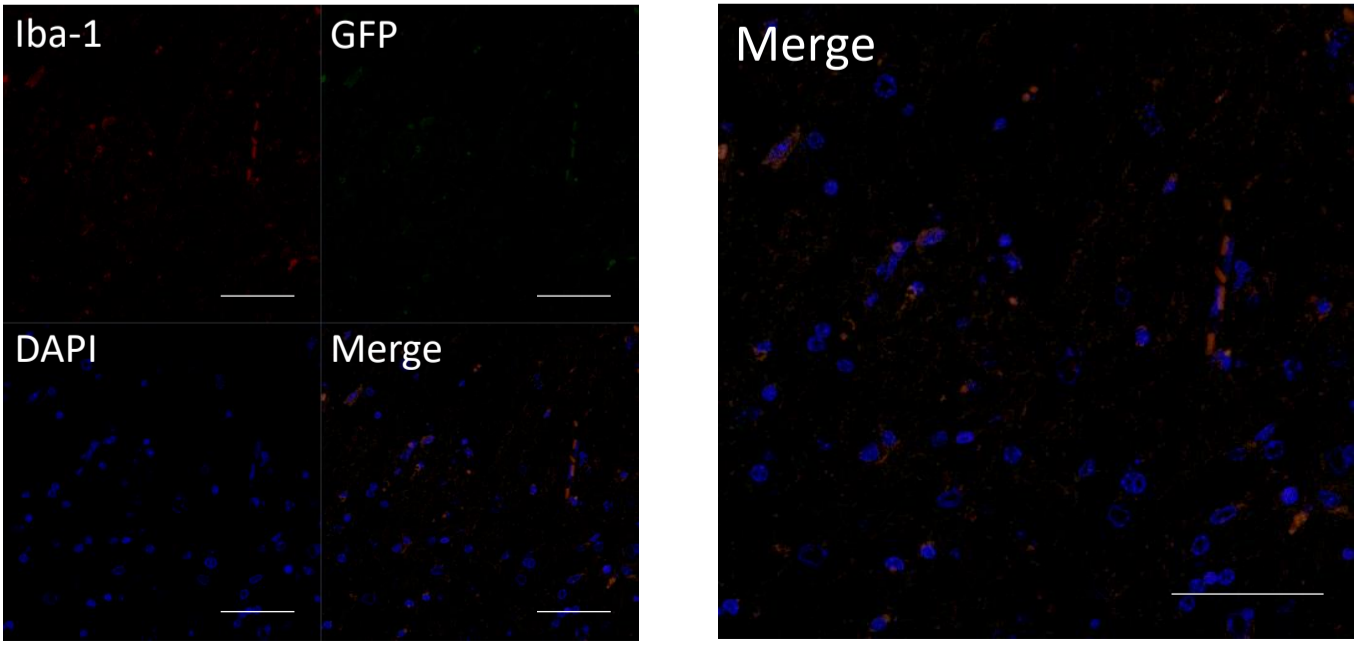

Supplement: S1 Fig — The separate channels for each image are shown without alterations of contrast or brightness and no cropping (Scale bar = 50μm). (PDF) [file pone.0262755.s001.pdf]
